# Interpetrosal sphingosine-1-phosphate ratio predicting Cushing’s disease tumor laterality and remission after surgery

**DOI:** 10.3389/fendo.2023.1238573

**Published:** 2023-10-31

**Authors:** Heng Sun, Chunli Wu, Biao Hu, Yuan Xiao

**Affiliations:** ^1^ Department of Endocrinology, Endocrinology Research Center, Xiangya Hospital of Central South University, Changsha, Hunan, China; ^2^ National Clinical Research Center for Geriatric Disorders, Xiangya Hospital of Central South University, Changsha, Hunan, China

**Keywords:** ipss, sphingosine-1-phosphate, Cushing’s disease, remission, tumor laterality

## Abstract

**Background:**

Cushing’s disease (CD) poses significant challenges in its treatment due to the lack of reliable biomarkers for predicting tumor localization or postoperative clinical outcomes. Sphingosine-1-phosphate (S1P) has been shown to increase cortisol biosynthesis and is regulated by adrenocorticotropic hormone (ACTH).

**Methods:**

We employed bilateral inferior petrosal sinus sampling (BIPSS), which is considered the gold standard for diagnosing pituitary sources of CD, to obtain blood samples and explore the clinical predictive value of the S1P concentration ratio in determining tumor laterality and postoperative remission. We evaluated 50 samples from 25 patients who underwent BIPSS to measure S1P levels in the inferior petrosal sinuses bilaterally.

**Results:**

Serum S1P levels in patients with CD were significantly higher on the adenoma side of the inferior petrosal sinus than on the nonadenoma side (397.7 ± 15.4 *vs*. 261.9 ± 14.88; P < 0.05). The accuracy of diagnosing tumor laterality with the interpetrosal S1P and ACTH ratios and the combination of the two was 64%, 56% and 73%, respectively. The receiver operating characteristic curve analysis revealed that the combination of interpetrosal S1P and ACTH ratios, as a predictor of tumor laterality, exhibited a sensitivity of 81.82% and a specificity of 75%, with an area under the curve value of 84.09%. Moreover, we observed that a high interpetrosal S1P ratio was associated with nonremission after surgery. Correlation analyses demonstrated that the interpetrosal S1P ratio was associated with preoperative follicle-stimulating hormone (FSH), luteinizing hormone (LH), and postoperative ACTH 8 am levels (P < 0.05).

**Conclusion:**

Our study demonstrated a significant association between the interpetrosal S1P ratio and tumor laterality, as well as postoperative remission in CD, suggesting that the interpetrosal S1P ratio could serve as a valuable biomarker in clinical practice.

## 1 Introduction

Cushing’s disease (CD), also known as adrenocorticotropic hormone (ACTH)-secreting pituitary adenoma, arises from the pituitary corticotroph cells and induces endogenous hypercortisolism by stimulating the adrenal glands to produce excessive amount of cortisol (1). Patients with CD typically exhibit symptoms of hypercortisolism, such as hypertension, diabetes, purplish skin striae, mental disturbances, hyposexuality, hirsutism, menstrual disorders, acne, fatigue, obesity, and osteoporosis (1). The overall mortality of patients with CD is twice that of the general population, and if left untreated, hypercortisolism resulting from CD increases this rate to approximately four times the expected value (2–4). Transsphenoidal surgery continues to be the primary treatment for CD (5). However, previous studies reported variable remission rates, ranging from 45% to 95% (6–8). Long-term follow-up data have revealed recurrence in 3–66% of patients who had initially achieved complete remission (9, 10). The rate of surgical remission in CD can be influenced by various factors, including the size and location of the tumor, expertise of the neurosurgeon, and criteria used for assessing remission (11). Preoperative clinical variables, such as age, gender, disease duration, and severity of clinical signs and symptoms, cannot reliably identify patients at a higher risk of nonremission (12, 13). Therefore, predicting postsurgical remission in CD remains a challenging goal.

Accumulating evidence has shown that sphingosine-1-phosphate (S1P), an intracellular pleiotropic bioactive sphingolipid metabolite synthesized by sphingosine kinase 1 (SPHK1), plays a pivotal role in diverse endocrine disorders (14–16). Overexpression of SPHK1 promotes the progression of multiple neuroendocrine tumors (17, 18). ACTH can rapidly activate sphingolipid metabolism, causing an increase in S1P secretion in the adrenal cortex (19). Furthermore, the activation of S1P signaling in H295R cells, a human adrenocortical tumor cell line, has been suggested to induce increased transcription of hormone-sensitive lipase and steroidogenic acute regulatory protein, ultimately elevating cortisol production (20). Recently, surgical removal of ACTH-secreting adenoma has been reported to cause a decline in sphingomyelin levels (21). However, whether they have a similar role in the pituitary gland remains to be investigated.

Bilateral inferior petrosal sinus sampling (BIPSS) is a highly effective procedure for diagnosing pituitary sources of ACTH in CD (22, 23). Contemporaneous differences in ACTH concentration during venous sampling between the two sides of the adenoma can predict the location of the adenoma within the pituitary (on the side of the gland with a microadenoma) and may guide surgical treatment in cases with inconclusive magnetic resonance imaging findings. Previous studies demonstrated that an ACTH gradient of ≥1.4 between the inferior petrosal sinuses can indicate microadenoma lateralization in patients with CD (24–26). However, the correct lateralization only occurs in 57–68% of all cases (27–29).

Therefore, we analyzed the clinical behavior of a well-characterized cohort of patients with CD who underwent BIPSS before surgery. We measured the difference in the concentration of S1P in bilateral petrosal sinus blood samples and explored the clinical predictive value of the S1P concentration ratio in determining tumor laterality and postoperative remission.

## 2 Materials and methods

### 2.1 Patients and study design

This study was conducted at a tertiary center, involving a cohort of 25 patients diagnosed with CD who had undergone BIPSS and surgery, with a minimum follow-up duration of 2 years. Comprehensive chart reviews were conducted to collect data on demographics, clinical characteristics, pituitary imaging findings, tumor pathology, and biochemical tests.

The criteria used for diagnosing CD encompassed the presence of characteristic signs and symptoms of hypercortisolism, along with biochemical evaluation of two urinary free cortisol measurements exceeding the normal range for the respective assay, serum cortisol level >1.8 μg/dL (50 nmol/L) after an overnight 1-mg dexamethasone suppression test, and two late-night salivary cortisol measurements exceeding the normal range for the respective assay (30). A diagnosis of Cushing’s syndrome was established if the patient had positive test results for at least two of the three aforementioned tests. Adrenal insufficiency was diagnosed if patients exhibited symptoms or signs of adrenal insufficiency or if serum cortisol levels were ≤3 μg/dL, even in the absence of clinical signs or symptoms. Remission was defined as normalization of the levels of 24-h urinary free cortisol, late-night salivary cortisol, and overnight 1-mg dexamethasone suppression test in patients without concurrent central adrenal insufficiency after surgery (31).

### 2.2 Patients and tissue/serum samples

Surgical specimens of CD-affected tissues were collected from Xiangya Hospital, Central South University. Three normal pituitary tissues were obtained from cadaveric organ donors without any history of endocrine disease (Central South University). A total of 25 CD tissue samples were obtained for immunohistochemistry analysis. This study was conducted in compliance with the Helsinki Declaration and was ethically approved by the Xiangya Hospital Ethics Committee, Xiangya Hospital (Changsha, China). Tumor samples and corresponding clinical materials were obtained with written consent from all patients.

### 2.3 BIPSS

After obtaining informed consent, BIPSS was performed using standard techniques described in previous studies (32, 33). Briefly, the patient’s head was immobilized to ensure midline positioning and prevent any potential bias towards asymmetric pituitary drainage by the petrosal sinuses. After placing peripheral catheters and cannulating both inferior petrosal sinuses, blood samples were collected at baseline and at 3, 5, 10, and 15 min following intravenous administration of DDAVP, which stimulates pituitary production of ACTH. Additional samples for experimental purposes were collected immediately following the 15-min sample collection to avoid interference with the patient’s diagnostic study.

### 2.4 Measurement of baseline plasma S1P concentration

Blood samples were obtained from both petrosal sinuses and were centrifuged to remove cellular components. Samples that exhibited hemolysis or coagulation were excluded from the study. Plasma samples were stored at −80°C. The S1P levels in plasma were analyzed using a S1P competitive ELISA kit (Echelon Biosciences, Salt Lake City, UT) according to the manufacturer’s instructions (34).

### 2.5 Immunofluorescence staining

The pituitary tissues were post-fixed and dehydrated with alcohol as follows: 70% for 24 h, 80% for 3 h, 90% for 4 h, 95% for 3 h, and finally in absolute alcohol for 2 h. Tissue slices with a 5-μm thickness were cut using a microtome (Thermo Fisher Scientific), blocked with 3% BSA, and then treated with primary antibodies to SPHK1 (CST, #3297) and ACTH (Proteintech, CL488-66358). Subsequently, the tissue slides were incubated with Alexa Fluor 488-conjugated anti-rabbit (Invitrogen, A21206, 1:200) or Alexa Fluor 555-conjugated anti-rabbit (Invitrogen, A21428, 1:200) secondary antibodies. Specimens were visualized and imaged using a fluorescence microscope.

### 2.6 Statistical analysis

The Mann–Whitney U test was used to assess the clinical–molecular associations in adenoma samples, whereas the chi-square test was used to compare categorical data. The Kruskal–Wallis analysis and ANOVA were conducted for multiple comparisons. Statistical analyses were conducted using SPSS v20 and GraphPad Prism version 7. All results were presented in graphs and tables as median ± interquartile range. The distribution of each parameter was presented as the minimum–maximum range. Parametric or nonparametric statistical tests were applied, as appropriate, after testing for normality. The receiver operating characteristic curve was used to determine the cut-off value for predicting tumor laterality. Pearson correlation analyses was used to examine the correlations between variables. Proportions were expressed as percentages, and significance was defined as P < 0.05.

## 3 Results

### 3.1 Clinical characteristics of remission and nonremission in patients with CD

**Figure 1:**
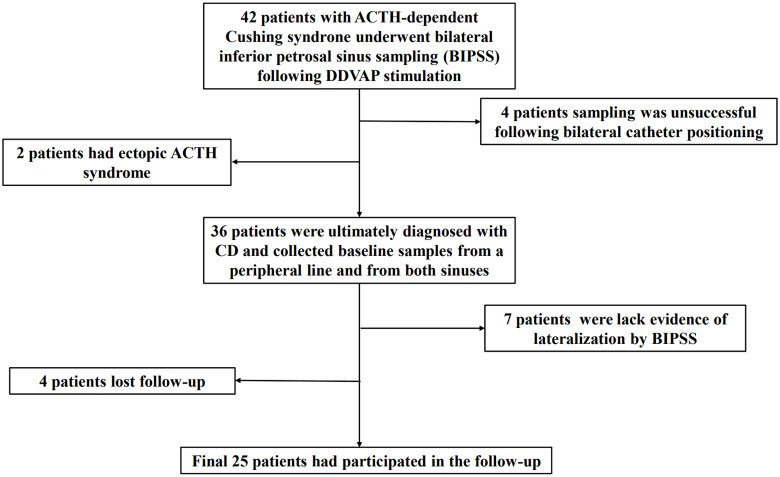
Flowchart of the screening process employed to select eligible participants for the study.

This study included 25 patients with CD who underwent BIPSS before surgery (**Figure 1**). Among them, 12 patients had microadenomas, whereas the remaining 13 had inconclusive magnetic resonance imaging findings; clinicopathological data are summarized in **Supplementary Table 1**. **Table 1** displays the demographics of patients who achieved remission (n = 16) and those who did not (n = 9). No significant differences were observed in terms of sex, age at diagnosis, or radiological variables between patients who achieved and those who did not achieve remission (P > 0.05). Patients who achieved remission exhibited a higher prevalence of emotional lability (P < 0.05). However, no significant differences were observed in other parameters (P > 0.05).

**Table 1:** Baseline clinical features of patients with pituitary tumors secreting adrenocorticotropin.

Clinical characteristics	Nonremission (n = 9)	Remission (n = 16)	*P*
Sex (M/F)	1/8	3/13	.542
Age at diagnosis, y	38 ± 10	35 ± 15	.622
Preoperative BMI,	30.22 ± 8.99	29.8 ± 12.2	.678
Sign and symptoms			
Central obesity	8 (88.9%)	15(93.8%)	.182
Full-moon face	8(88.9%)	12(80.0%)	.356
Hirsutism	6(66.7%)	12(80.0%)	.566
Skin purple lines	5(55.6%)	9(56.3%)	.403
Capillary fragility	3(33.3%)	5(31.3%)	.645
Depression	1(11.1%)	4(25.0%)	.356
Emotional lability	3(33.3%)	13(81.3%)	.019
Irregular menstrual cycles	7(77.8%)	13(81.3%)	.085
Complications			
Hypertension	7(77.8%)	9(56.3%)	.088
Diabetes	2(22.2%)	5(31.3%)	.12
Radiological variables			
Macro-/Microadenoma	0/9	0/16	.98

Between-group comparison according to remission after first surgery.

Several recent studies have established morning cortisol level measured on postoperative day 1 (POD1) as a predictive biomarker for long-term remission of CD (35, 36). For biochemical features, patients who did not achieve remission exhibited higher serum cortisol (19.16 ± 5.55 *vs*. 5.95 ± 1.42; P = 0.014) and median serum (8 am) ACTH (10.26 ± 8.24 *vs*. 5.15 ± 3.68; P = 0.042) levels on POD1. No significant differences were observed in the preoperative baseline 4 pm serum cortisol levels, preoperative baseline 0 am serum cortisol levels, preoperative 8 pm ACTH levels, 4 pm ACTH levels, and 0 am ACTH levels (P > 0.05) (**Table 2**). In addition preoperative FT3, FT4, TSH, GH, FSH, LH, and PRL levels were comparable in patients with and without remission.

**Table 2:** Baseline clinical and biochemical features of patients with pituitary tumors secreting adrenocorticotropin.

	Nonremission (n = 9)	Remission (n = 16)	*P*
Biochemical variables			
Presurgery baseline 8am cortisol, µg/dL	24.61 ± 9.32	21.50 ± 6.80	.352
Presurgery baseline 4pm cortisol, µg/dL	18.59 ± 7.66	14.47 ± 5.31	.126
Presurgery baseline 0am serum cortisol, median, µg/dL	22.24 ± 9.39	15.58 ± 7.30	.060
Presurgery 24-h urinary cortisol, µg/24h	313 ± 380.56	277.42 ± 190.41	.072
Presurgery serum 8am ACTH, median, pg/mL	25.79 ± 14.12	24.56 ± 20.53	.875
Presurgery serum 4pm ACTH, median, pg/mL	19.46 ± 9.66	21.71 ± 15.31	.695
Presurgery serum 0am ACTH, median (IQR), pg/mL	18.94(10.91-24.66)	16.31(11.58-19.77)	.934
POD1 serum 8am serum cortisol, µg/dL	19.16 ± 5.55	5.95 ± 1.42	**.014**
POD1 serum 8am ACTH, pg/mL	10.26 ± 8.24	5.15 ± 3.68	**.042**

IQR interquartile range, POD1, postoperative day 1, S1P, Sphingosine 1-phosphate; ACTH, adrenocorticotropic hormone. Between-group comparison according to remission after first surgery.The bold values provided means the significant meaning in statistics because their P values are less than 0.05.

### 3.2 Overexpression of SPHK1 and higher concentrations of serum S1P on the tumor side in patients with CD

**Figure 2:**
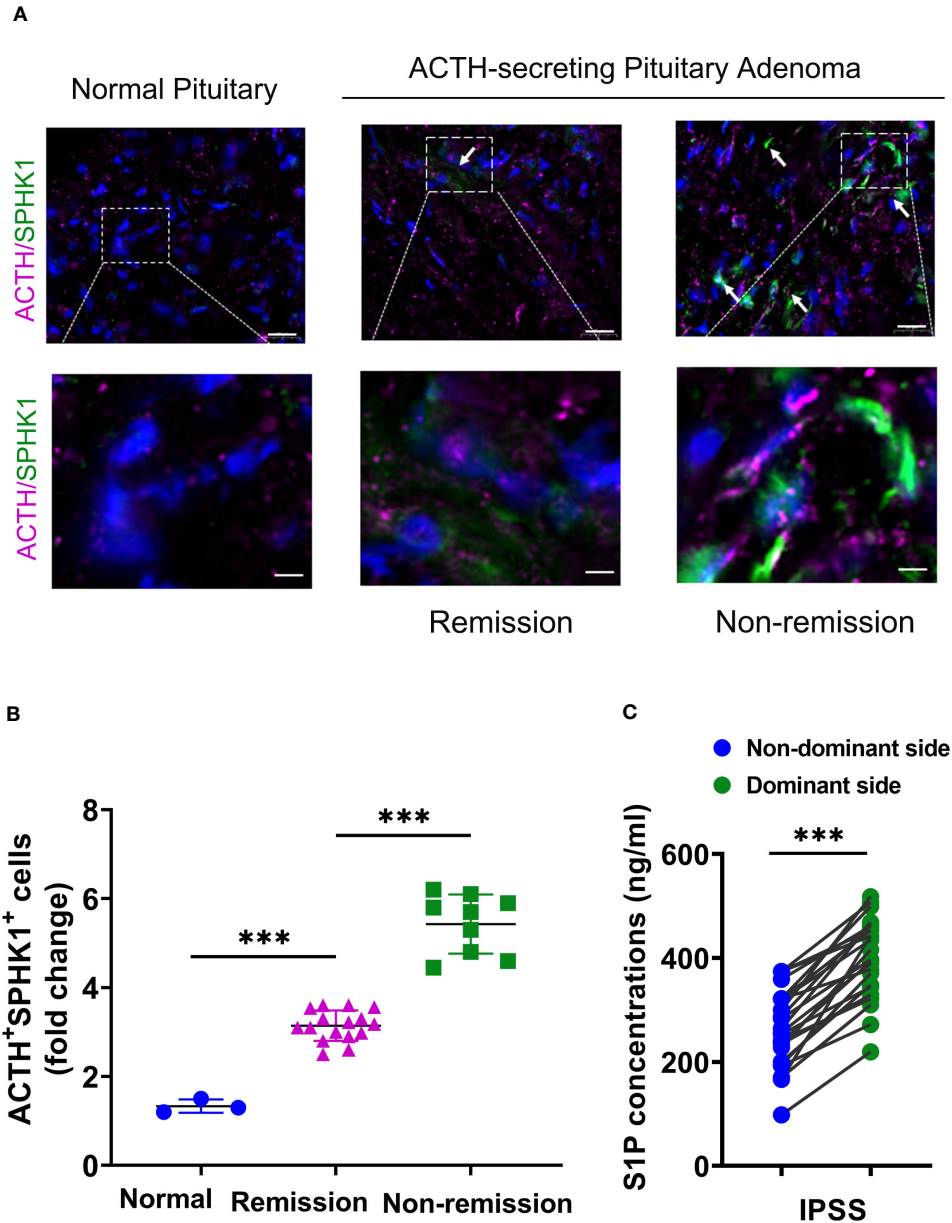
**(A)** Representative images of immunofluorescence double staining for SPHK1 (green) and ACTH (pink) in normal pituitary glands and ACTH-secreting pituitary adenomas from the remission and nonremission groups (Normal: n = 3, ACTH pituitary adenoma: remission *vs.* nonremission: n = 16 *vs.* 9); scale bars: 100-μm upper and 50-μm lower. **(B)** Quantitative analysis; white arrows indicate double-positive cells for ACTH and SPHK1. **(C)** The concentration of S1P in the plasma obtained from the inferior petrosal sinus of the adenoma side and nonadenoma side. ***P < 0.001. Bar represents mean ± SD.

Prior studies have demonstrated that ACTH acutely activates SPHK1 to increase S1P concentrations (19). Upregulation of SPHK1 is associated with poor prognosis in endocrine-related cancer (17, 18, 21). To investigate the role of SPHK1 in CD, we performed a heatmap analysis of key genes involved in phospholipid metabolism and signaling pathways in CD adenomas and surrounding normal tissues using the GEO dataset (GEO208107). This analysis revealed the activation of crucial genes involved in phospholipid metabolism and signaling pathways in ACTH-secreting pituitary adenomas (**Supplementary Figure 1**). Subsequently, we compared the association between pituitary SPHK1 expression and proopiomelanocortin, corticotropin-releasing hormone, corticotropin releasing hormone receptor 1, and corticotropin releasing hormone receptor 2 in pituitary tumor tissues and identified a positive correlation between SPHK1 and ACTH tumor-related genes in the TNM plot database (**Supplementary Figure 2**). To investigate the potential role of SPHK1 in CD, we compared the expression values of SPHK1 in the normal pituitary tissues and those obtained from patients with CD in the remission/nonremission groups. Immunofluorescence staining (**Figures 2A, B**; **Supplementary Figure 3**) revealed an increased number of double-positive cells for SPHK1 and ACTH in CD-affected pituitary tissues than those in the normal pituitary tissues. Furthermore, the proportion of double-positive cells for SPHK1 and ACTH was significantly higher in the nonremission CD adenomas tissues than that in the remission CD adenomas. Furthermore, we investigated the concentration of S1P in bilateral petrosal sinus blood samples and observed that the concentration was significantly higher on the adenoma side than that on the nonadenoma side (397.7 ± 15.4 *vs*. 261.9 ± 14.88; P < 0.05, **Figure 2C**). Thus, these findings suggested a close association between S1P concentration and the development of ACTH-secreting tumor.

### 3.3 Combination of interpetrosal S1P and ACTH ratios improved the diagnostic performance for adenoma laterality

**Figure 3:**
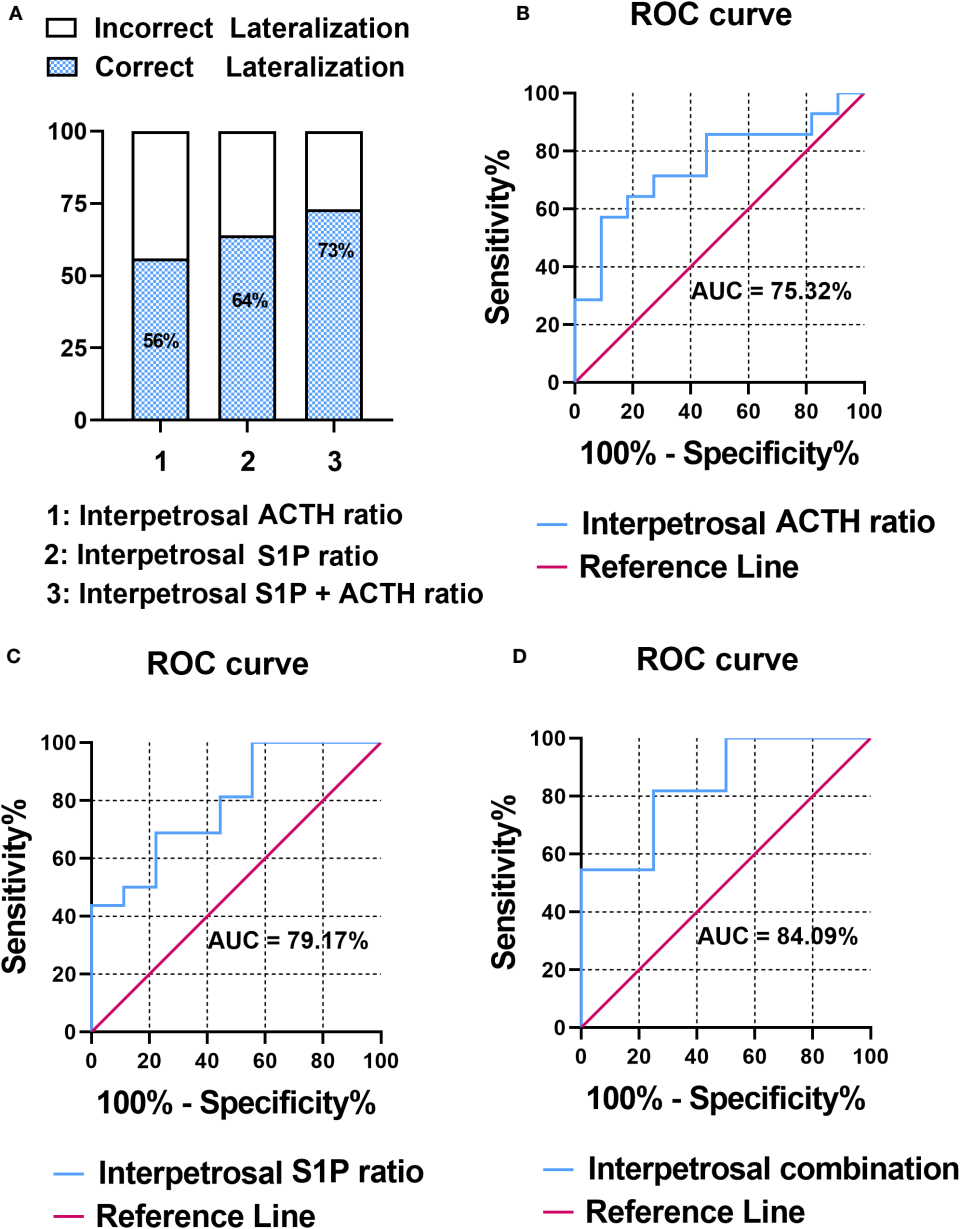
**(A)** Bar graph illustrating the accuracy of predicting tumor laterality. **(B)** Receiver operating characteristic (ROC) curve analysis of interpetrosal ACTH ratio to predict tumor location. **(C)** ROC curve analysis of the interpetrosal S1P ratio to predict tumor location. **(D)** ROC curve analysis of the combination of the interpetrosal S1P and ACTH ratios to predict tumor location.

The pathology of patients with CD was classified based on adenomatous tissue with ACTH-positive immunostaining into adenoma or nonadenoma sides. To evaluate the correlation between the interpetrosal S1P ratio lateralization and tumor location, we compared the accuracy of predicting tumor laterality using the interpetrosal S1P ratio (>1) and interpetrosal ACTH ratio (>1.4) (the interpetrosal ACTH ratio >1.4 is acknowledged for its positive role in predicting tumor laterality), as well as their combination. Our results indicated that using the interpetrosal S1P or ACTH ratios alone yielded accuracies of 64% and 56% respectively. Notably, the combination of both demonstrated a significantly improved accuracy of 73% (**Figure 3A**).

Thereafter, the receiver operating characteristic analysis was performed to determine the role of predicting tumor laterality. In particular, the interpetrosal ACTH ratio with an AUC of 75.32% (95% CI: 60.06–97.46%, P < 0.05) and the interpetrosal S1P ratio demonstrated a clinically significant diagnostic accuracy for lateralization, with an AUC of 79.17% (95% CI: 44.40–85.84%, P < 0.05). Furthermore, combining the interpetrosal S1P and ACTH ratios generated an receiver operating characteristic curve with an AUC of 84.09% (95% CI: 52.3–96.77%, P < 0.05) for predicting lateralization with tumor location (cutoff value: interpetrosal S1P ratio ≥1.06, interpetrosal ACTH ratio ≥2.8, 81.82% sensitivity, and 75% specificity) (**Figures 3B–D**).

### 3.4 Interpetrosal S1P ratio serves as a predictive factor for early remission in CD

**Figure 4:**
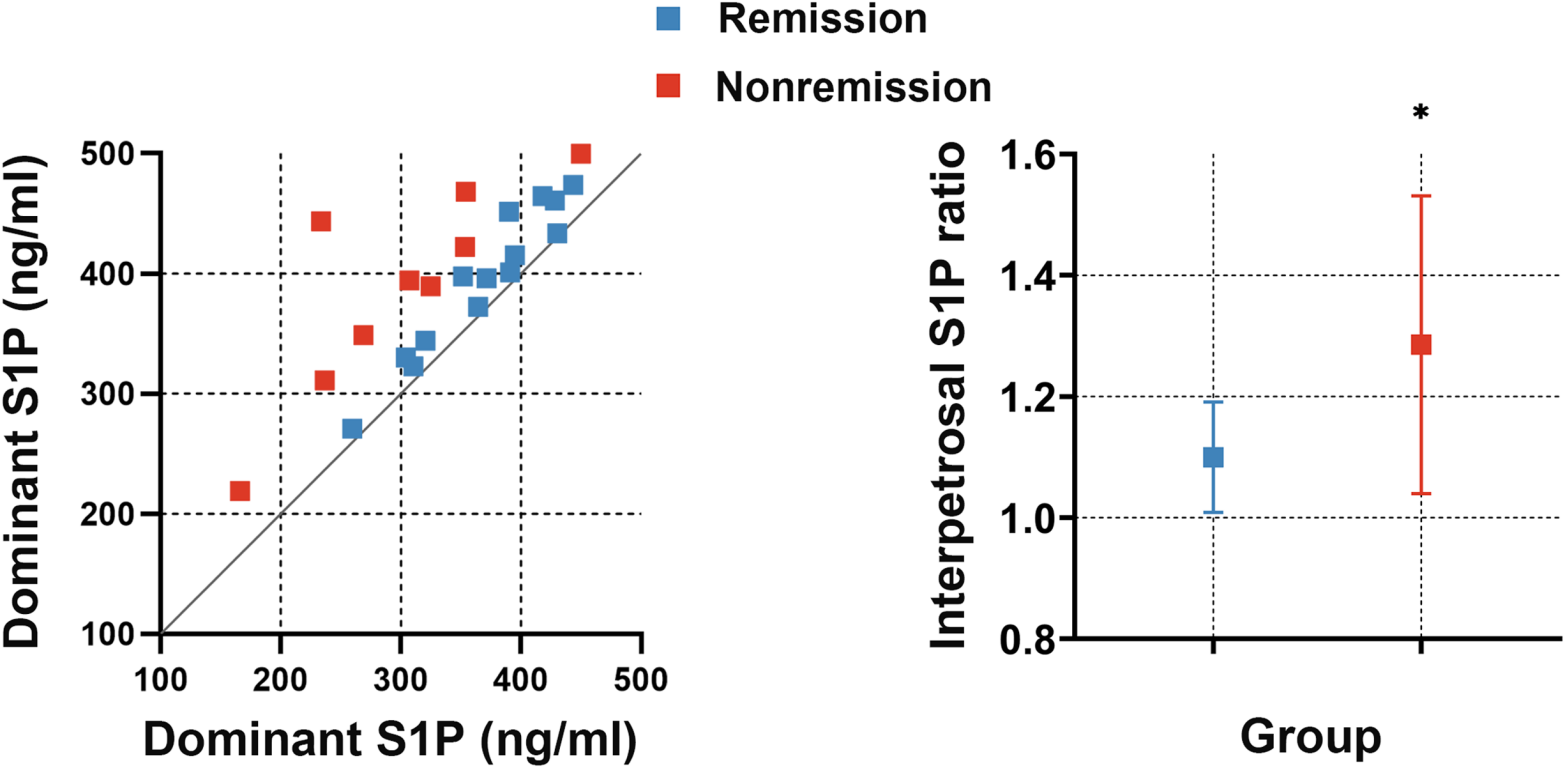
Left picture: Scatter plot of bilateral S1P concentrations in the remission and nonremission groups; the slope represents the interpetrosal S1P ratio, blue dots represent the remission group, and red dots represent the nonremission group. Right picture: The interpetrosal S1P ratio in the remission and nonremission groups. *P < 0.05. Bar represents mean ± SD.

To investigate whether the interpetrosal S1P ratio is associated with early postoperative remission in CD, we compared the baseline interpetrosal S1P ratio between patients with CD in the remission and nonremission groups. Interestingly, we observed that the nonremission group exhibited higher interpetrosal S1P ratios than those of the remission group (median, 1.28 ± 0.25 *vs*. 1.10 ± 0.09, P = 0.012) (**Figure 4**).

To investigate potential factors affecting the interpetrosal S1P ratio, we compared the correlation between interpetrosal S1P ratio and various clinical indicators. This analysis revealed that the interpetrosal S1P ratio positively correlated with preoperative FSH and LH levels, as well as with postoperative 8 am ACTH levels. No significant difference was observed between the interpetrosal S1P ratio and other indicators (**Supplementary Figure 4**).

## 4 Discussion

The use of BIPSS involves collection of samples from each inferior petrosal sinus simultaneously, enabling a direct comparison of ACTH concentrations between the left and right petrosal sinuses. BIPSS is used for two purposes: 1) to assist in the differential diagnosis of Cushing’s syndrome; and 2) to determine which side of the pituitary gland contains an adenoma in patients with CD. The interpetrosal ACTH ratio is also useful in determining the location/lateralization of pituitary microadenomas (24, 30, 37), thereby providing guidance to the neurosurgeon during surgery.

To our knowledge, this is the first study to demonstrate that serum S1P levels in patients with CD are significantly higher on the adenoma side of the inferior petrosal sinus than on the nonadenoma side. The interpetrosal S1P ratio exhibited a positive significance in predicting tumor laterality, and the predictive performance was improved when S1P was combined with the interpetrosal ACTH ratio. Notably, the interpetrosal S1P ratio exhibited a positive significance in predicting remission after surgery. Furthermore, the interpetrosal S1P ratio demonstrated a positive and significant correlation with preoperative FSH and LH levels, as well as 8 am ACTH levels on POD1.

ACTH is recognized for its role in controlling the expression of genes involved in steroid production and cortisol synthesis in the human adrenal cortex through sphingolipid metabolism (19). Specifically, ACTH rapidly stimulates SPHK1 activity, leading to an increased in S1P levels, which in turn, increases the expression of multiple steroidogenic proteins (20). Our study demonstrated that higher S1P concentrations were present on the tumor side than on the nontumor side in patients with CD, indicating that the regulatory relationship between ACTH and S1P also exists in ACTH-secreting pituitary adenomas. Several pieces of evidence have supported the potential relationship between S1P and the occurrence of CD. Interestingly, SPHK1 and S1P are known to be integral to the regulation of epidermal growth factor receptor (EGFR) (38), which is highly expressed in human corticotropinomas, where it triggers proopiomelanocortin (the precursor of ACTH) transcription and ACTH synthesis (39). Blocking EGFR activity with an EGFR inhibitor can attenuate corticotroph tumor cell proliferation (40). Furthermore, SPHK1 and proopiomelanocortin share a common transcriptional coactivator, P300 (41, 42). Notably, S1P also directly binds to and inhibits histone deacetylase 2, thereby regulating histone acetylation and gene expression (43). Notably, histone deacetylase 2 expression is deficient in ACTH-pituitary adenomas in CD, contributing to glucocorticoid insensitivity (44), which is a hallmark of CD and a feature associated with nonremission. These studies further demonstrated an association between high S1P ratio and nonremission of CD. Our study, for the first time, established an association between SPHK1/S1P and ACTH adenoma. Nevertheless, further experimental verification is required to confirm the existence of common pathways linking SPHK1 and ACTH. Thus, these findings indicated that the S1P ratio can, to some extent, reflect the differences in ACTH levels and may serve as a surrogate marker for detecting ACTH-secreting pituitary adenomas.

BIPSS is a highly effective procedure for diagnosing pituitary sources of ACTH in CD and remains the gold standard diagnostic method. However, some findings indicated certain limitations associated with the use of the inferior petrosal sinus sampling (IPSS) method in predicting tumor lateralization. The possible causes of error include asymmetrical or underdeveloped petrosal sinus anatomy and placement of the catheter (27). The present study revealed a notable increase in the interpetrosal ACTH ratio among patients with accurate predictions of tumor laterality than among those with inaccurate predictions, although the positive predictive value remained low. These findings suggested that other mechanisms may exist that contribute to false-positive results. The limitations on lateralization highlighted the need for further research to understand the underlying mechanisms contributing to the accuracy of IPSS in predicting tumor lateralization. Further investigation is required to understand these potential mechanisms and improve the accuracy of IPSS in predicting tumor lateralization.

We observed that the interpetrosal S1P ratio was slightly more effective than the ACTH ratio in predicting tumor laterality. However, combining both methods significantly improved the diagnostic sensitivity and specificity. These results have important implications for clinical practice as accurate tumor lateralization is essential for the correct management and treatment of pituitary adenomas. Overall, these findings highlighted the importance of using multiple measures in predicting tumor lateralization and suggested that combining measures may be more effective than relying on any single measure alone. Future research should investigate additional measures to improve the accuracy of tumor lateralization and optimize the use of existing measures for making clinical decisions.

The initial treatment recommendation for CD is surgery. However, long-term surveillance is necessary because of the high recurrence rate (12). Therefore, identifying patients who are at a greater recurrence risk would be helpful in establishing an effective surveillance strategy. Our study revealed that the expression of SPHK1 in pituitary tissue was higher in postoperative nonremission group than in postoperative remission group. Moreover, patients in the nonremission group exhibited significantly higher interpetrosal S1P ratios than those of patients in the remission group. SPHK1 catalyzes the direct phosphorylation synthesis of S1P, and the S1P ratio can thus reflect the expression level of SPHK1 in ACTH tumors. Since S1P can increase the expression of multiple steroidogenic proteins, including steroidogenic acute regulatory protein, 18-kDa translocator protein, low-density lipoprotein receptor, and scavenger receptor class B type I (20), the interpetrosal S1P ratios may be indicative of disease prognosis. This finding is consistent with previous findings indicating the overexpression of SPHK1 is associated with poor prognosis in various neuroendocrine tumors, as factors associated with tumor proliferation, S1P and SPHK1, may play a key role in the proliferation and survival of ACTH pituitary adenomas. The high proportions of SPHK1/ACTH double-positive cells are likely associated with greater phenotypic severity, and CD tumors with this phenotype may have a poor prognosis. These findings hold clinically significance for predicting early postoperative remission in patients with CD. As aforementioned, the interpetrosal S1P ratios have been suggested as a useful diagnostic tool for determining adenoma lateralization in CD, which can also serve as a prognostic indicator for postoperative remission.

Pearson correlation analysis indicated that ACTH 8 am on POD1 and FSH/LH levels were significantly associated with the interpetrosal S1P ratio, suggesting that these pituitary dysfunctions may have a role in the early remission of CD. However, the sample size in this study was relatively small, and further studies with larger sample sizes are needed to confirm these findings. Additionally, other factors affecting surgical outcomes, such as the experience of the surgeon, extent of surgical resection, and use of adjuvant therapy, should be considered when predicting postoperative remission in patients with CD.

This study has some limitations. First, the study was retrospective in design, which limited the control of confounding factors. Additionally, because of the limited sample size, we did not specifically investigate cases where the ACTH ratio failed to accurately identify the correct tumor location. Finally, we did not explore the functional evidence of a common pathway between SPHK1 and ACTH. Despite these limitations, the study contributes to our understanding of the potential utility of the interpetrosal S1P ratio as a biomarker for CD and provides a basis for future research in this area.

In conclusion, our study demonstrated a significant association between the interpetrosal S1P ratio and tumor laterality, as well as in early remission in CD. These findings suggested that the interpetrosal S1P ratio could serve as a useful biomarker in clinical practice. Moreover, targeting genes and drugs related to SPHK1/S1P could provide novel therapeutic strategies for treating CD.

## Data availability statement

The original contributions presented in the study are included in the article/**Supplementary Material**. Further inquiries can be directed to the corresponding author.

## Ethics statement

The studies involving humans were approved by The Xiangya Hospital Ethics Committee, Xiangya Hospital (Changsha, China). The studies were conducted in accordance with the local legislation and institutional requirements. The participants provided their written informed consent to participate in this study.

## Author contributions

HS: conceptualization, methodology, software, visualization, and investigation. CW and BH: software. YX: writing – review & editing. All authors contributed to the article and approved the submitted version.
