# Supplementary material for: Interpetrosal sphingosine-1-phosphate ratio predicting Cushing’s disease tumor laterality and remission after surgery
Source: Front Endocrinol (Lausanne). 2023 Oct 31;14:1238573. doi: 10.3389/fendo.2023.1238573 (PMC10644774; doi:10.3389/fendo.2023.1238573)
Supplement: Supplementary file 1 [file DataSheet_1.docx]

Interpetrosal Sphingosine-1-Phosphate Ratio Predicting Cushing’s Disease Tumor Laterality and Remission after Surgery

Heng Sun ^a^, Chunli Wu ^a^, Biao Hu ^a^, Yuan Xiao ^a,b,*^

**Supplementary Table.1** Demographic and imaging features of patient population. Data are presented with findings at BIPSS and surgery

| **n** | **Age** | **Gen der** | **MRI** | **Lateralization  on interpetrosal S1P Ratio** | **Treatment** | **Pathology** |
| --- | --- | --- | --- | --- | --- | --- |
| 1 | 37 | F | Negative | Right | Tumor resection | Right adenoma |
| 2 | 36 | F | 4mm | Right | Tumor resection | Left adenoma |
| 3 | 38 | F | Subtle | Left | Tumor resection | Left adenoma |
| 4 | 31 | F | Subtle | Right | Tumor resection | Left adenoma |
| 5 | 58 | F | 5mm | Right | Tumor resection | Right adenoma |
| 6 | 28 | F | 7mm | Left | Tumor resection | Left adenoma |
| 7 | 10 | F | Subtle | Left | Tumor resection | Left adenoma |
| 8 | 40 | F | 6mm | Left | Tumor resection | Left adenoma |
| 9 | 38 | F | 4mm | Left | Tumor resection | Left adenoma |
| 10 | 41 | F | 4mm | Left | Tumor resection | Left adenoma |
| 11 | 35 | F | Negative | Right | Tumor resection | Right adenoma |
| 12 | 60 | F | Negative | Right | Tumor resection | Left adenoma |
| 13 | 47 | M | Subtle | Right | Tumor resection | Left adenoma |
| 14 | 39 | M | 5mm | Left | Tumor resection | Left adenoma |
| 15 | 52 | F | Subtle | Right | Tumor resection | Right adenoma |
| 16 | 19 | M | Negative | Right | Tumor resection | Right adenoma |
| 17 | 46 | F | 5mm | Right | Tumor resection | Right adenoma |
| 18 | 23 | F | 4mm | Right | Tumor resection | Right adenoma |
| 19 | 23 | F | 6mm | Right | Tumor resection | Left adenoma |
| 20 | 54 | F | 5mm | Right | Tumor resection | Right adenoma |
| 21 | 52 | F | 4mm | Right | Tumor resection | Left adenoma |
| 22 | 31 | F | Subtle | Left | Tumor resection | Left adenoma |
| 23 | 24 | M | Subtle | Right | Tumor resection | Left adenoma |
| 24 | 55 | F | Subtle | Right | Tumor resection | Left adenoma |
| 25 | 32 | F | Subtle | Right | Tumor resection | Left adenoma |

Subtle abnormalities: non-discrete lesions ≤ 4 mm. MRI negative: no adenoma was visible by either the neurosurgeon or the neuroradiologist. Lateralization on BIPSS: an intersinus gradient of > 1.4:1.

**
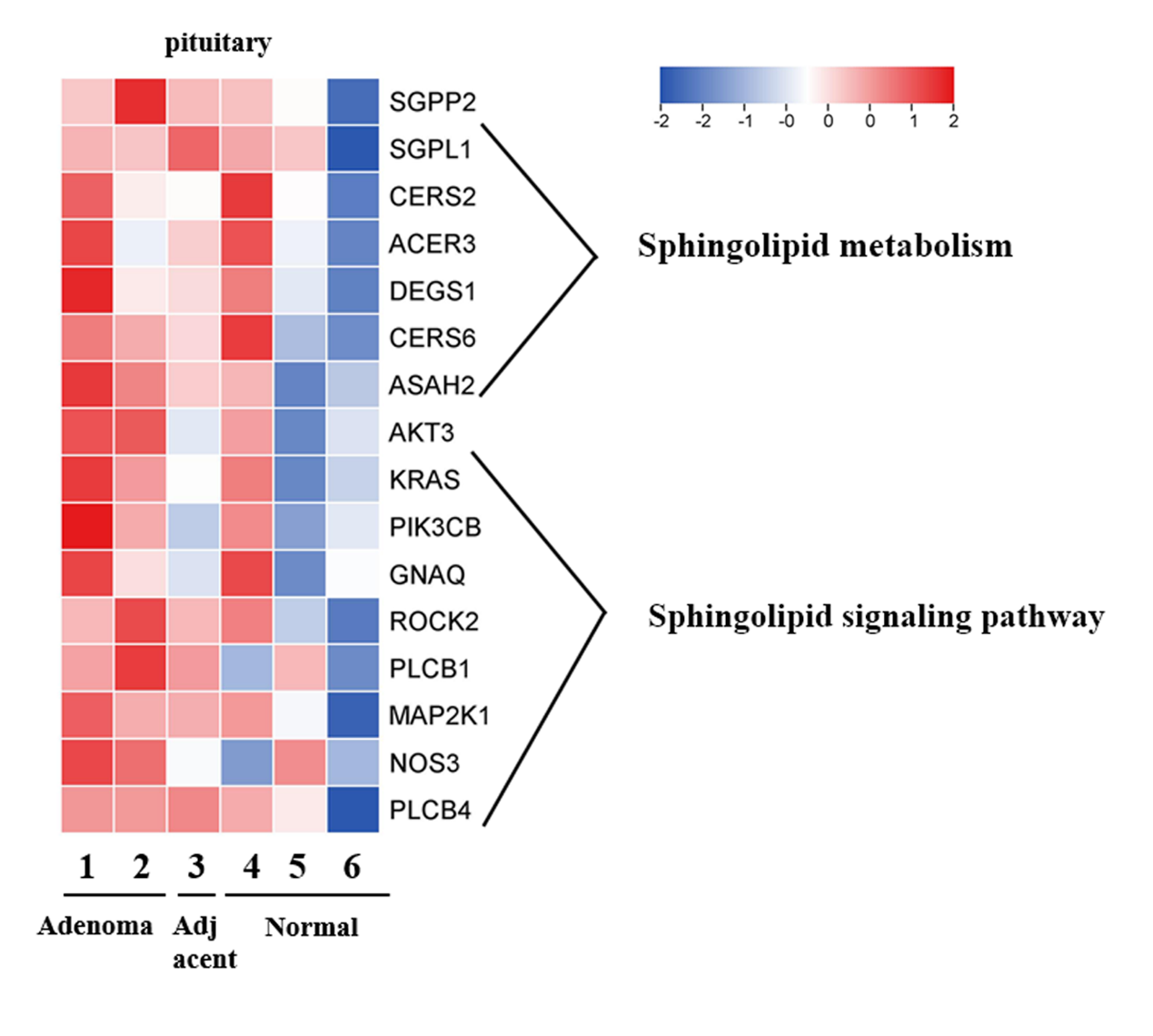
Supplementary Figure.1** Gene sets of Sphingolipid metabolism and sphingolipid signaling pathways. RNA-seq comparing Cushing's disease adenomas and surrounding normal tissues (GSE208107)**.** Adenoma group (number 1-2) represent group CD adenoma, Adjacent group (number 3) represent normal tissues surrounding CD adenomas, Normal group (number 4-6) represent normal human pituitary tissues.

**
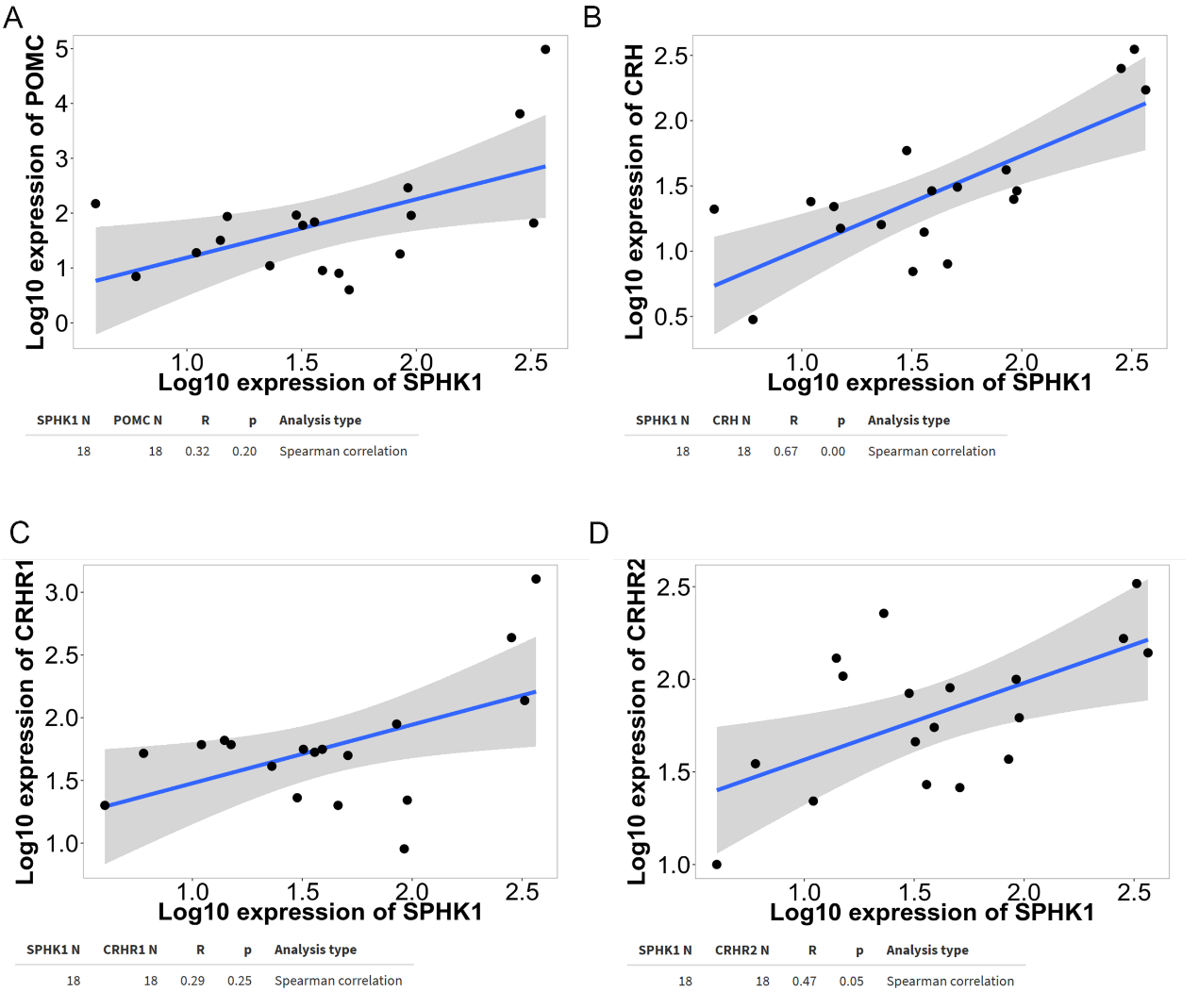
**

**Supplementary Figure.2** The correlation between the expression of POMC, CRH, CRHR1, CRH2 and SPHK1 in pituitary tumor tissues from TNM plot database.


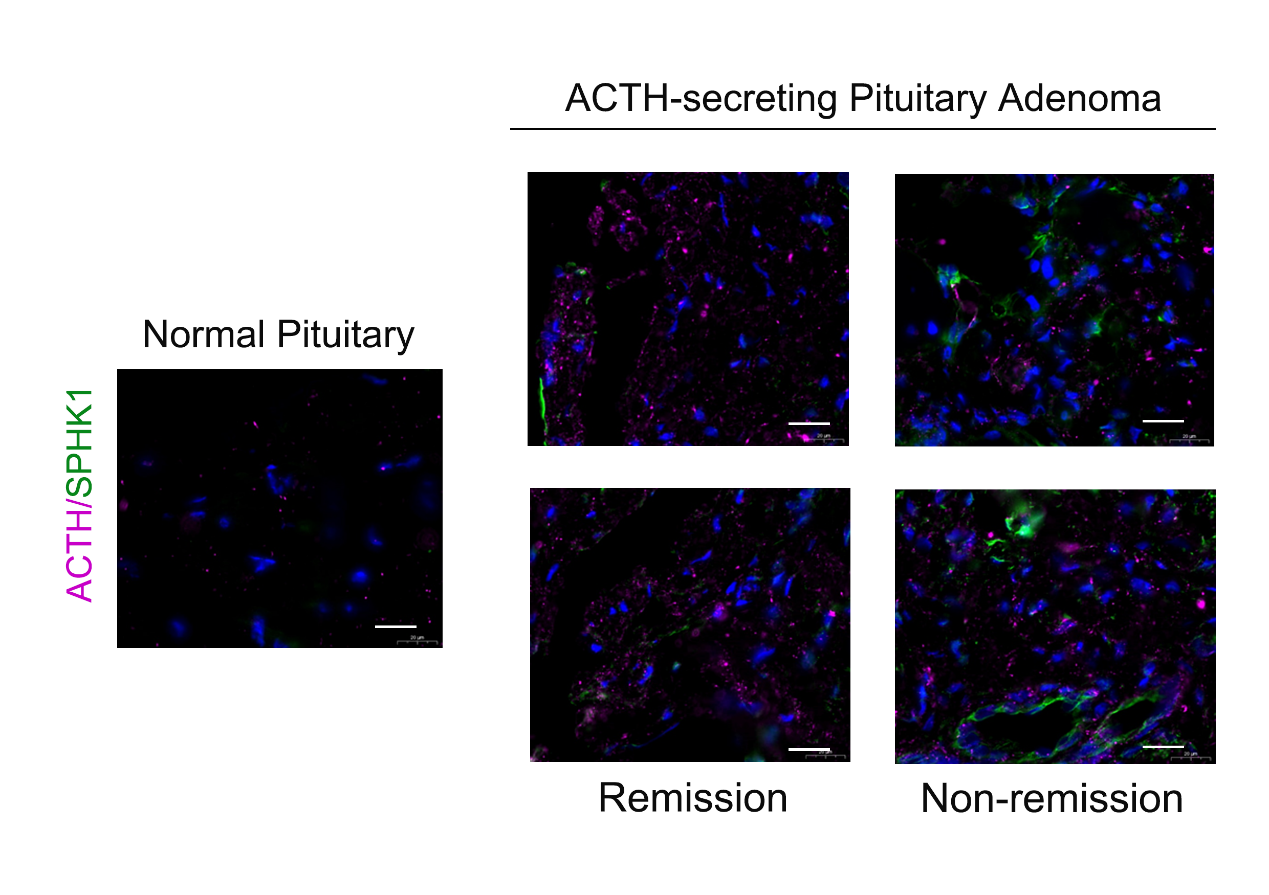
**Supplementary Figure.3** Several representative images of immunofluorescence (IF) double staining for SPHK1 (green) and ACTH (pink) in the normal pituitary gland and ACTH-secreting pituitary adenoma including remission and nonremission groups (Normal: n = 3, ACTH pituitary adenoma: remission vs nonremission: n = 16 vs 9;); Scale bars: 100 μm.


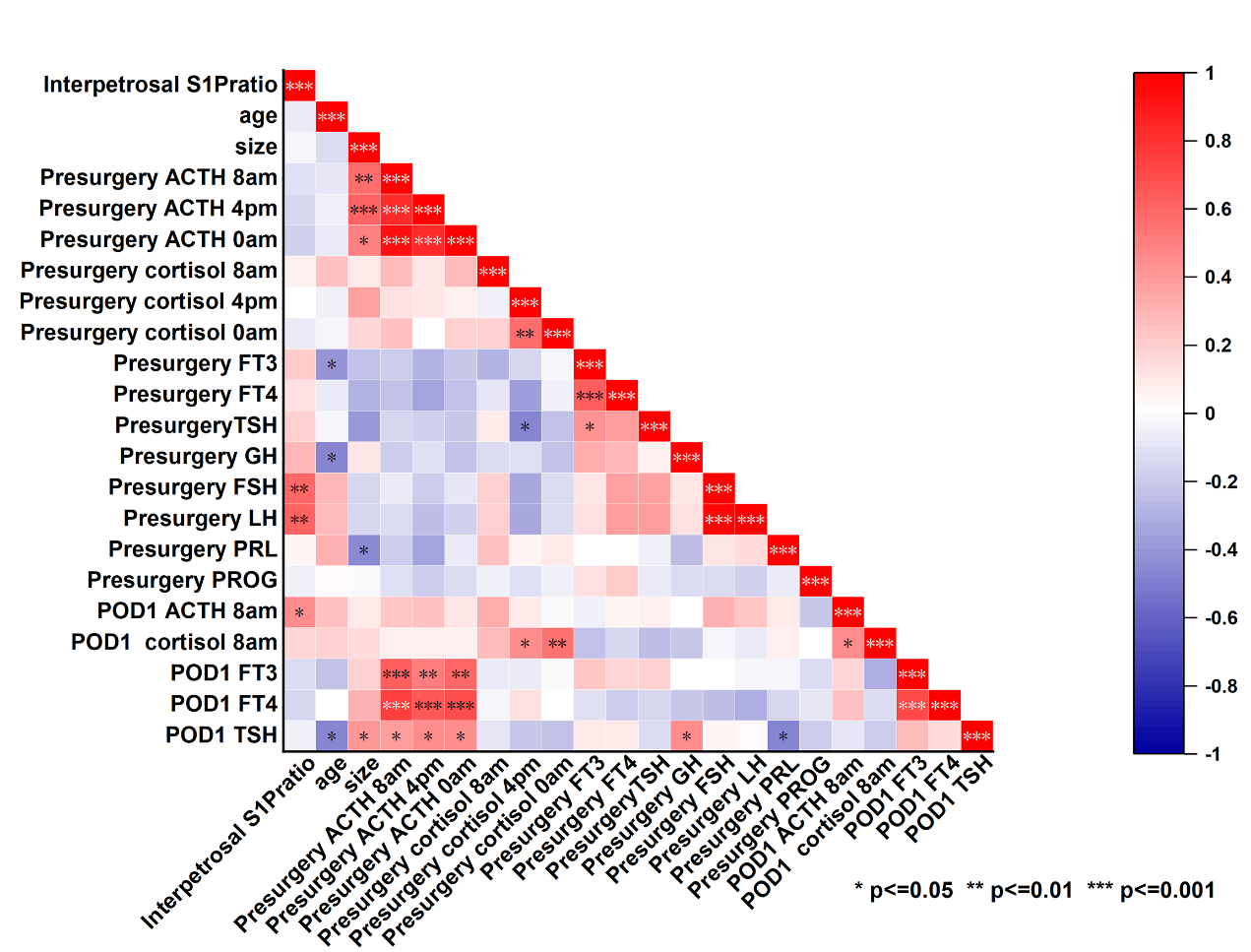


**Supplementary Figure.4** A heatmap of the Pearson correlation analysis between interpetrosal S1P ratio and other clinical variables.
